# Characterisation of Phantom Limb Pain in Traumatic Lower-Limb Amputees

**DOI:** 10.1155/2021/2706731

**Published:** 2021-12-13

**Authors:** André Tadeu Sugawara, Marcel Simis, Felipe Fregni, Linamara Rizzo Battistella

**Affiliations:** ^1^Instituto de Medicina Física e Reabilitação, Hospital das Clínicas HCFMUSP, Faculdade de Medicina, Universidade de São Paulo, São Paulo, SP, Brazil; ^2^Neuromodulation Center, Spaulding Rehabilitation Hospital, Harvard Medical School, Boston, MA, USA; ^3^Departamento de Medicina Legal, Ética Médica e Medicina Social e do Trabalho, Faculdade de Medicina FMUSP, Universidade de São Paulo, São Paulo, SP, Brazil

## Abstract

**Introduction:**

There is no diagnosis for phantom limb pain (PLP), and its investigation is based on anamnesis, which is subject to several biases. Therefore, it is important to describe and standardize the diagnostic methodology for PLP.

**Objective:**

To characterise PLP and, secondarily, to determine predictors for its diagnosis. *Methodology*. This is a cross-sectional study involving patients with unilateral traumatic lower-limb amputation aged over 18 years. Those with clinical decompensation or evidence of disease, trauma, or surgery in the central or peripheral nervous system were excluded. Sociodemographic and rehabilitative data were collected; PLP was characterised using the visual analogue scale (VAS), pain descriptors, and weekly frequency.

**Results:**

A total of 55 eligible patients participated in the study; most were male, young, above-knee amputees in the preprosthetic phase of the rehabilitation. The median PLP VAS was 60 (50–79.3) mm characterised by 13 (6–20) different descriptors in the same patient, which coexist, alternate, and add up to a frequency of 3.94 (2.5–4.38) times per week. The most frequent descriptor was movement of the phantom limb (70.91%). Tingling, numbness, flushing, itchiness, spasm, tremor, and throbbing are statistically significant PLP descriptor numbers per patient predicted by above-knee amputation, prosthetic phase, higher education level, and greater PLP intensity by VAS (*p* < 0.05).

**Conclusion:**

PLP is not a single symptom, but a set with different sensations and perceptions that need directed and guided anamnesis for proper diagnosis.

## 1. Introduction

Amputation causes a physical disability, easily and visually diagnosed, with immediate effects on functionality. Although it is restricted to situations where attempts to save the limb are unsuccessful or life threatening, it has high prevalence worldwide, with approximately 35.3 million people having lower-limb amputations. The ageing population, increasing poorly controlled comorbidities, violence, epidemics, traffic accidents, and other traumas may justify this high prevalence [1, 2].

Additionally, as an immediate effect on mobility, the amputee experiences pain in the residual limb and in the amputated part. Residual limb pain (RLP), sometimes called “stump pain,” is felt in the part that remains after an amputation and can be caused by bone, soft-tissue, bloody supply, or prosthesis fitting problems. On the other hand, phantom limb pain (PLP) is a pain in the missing part, defined by the International Association for the Study of Pain (IASP) as any sensation, movement, or posture; voluntary or involuntary; and perceived in the patient's missing body part and described as unpleasant. It excludes RLP and phantom limb sensations related to the nonunpleasant perception of the amputated (missing) part [3–5].

While RLP can be diagnosed by physical examination or imaging tests, the phantom limb cannot be examined, and the diagnosis is based on the patient's report, and on the way, the interviewer conducts the anamnesis [3–6]. There is great variability of the prevalence of PLP in amputees, with reports indicating a prevalence from 2% to 98% [2, 7–10]. Biological mechanisms are insufficient to explain this disparity, with cultural variations leading to different interpretations of the definition [11–15]. The anamnesis method used to investigate PLP may also lead to variations in interpretation. Many studies do not report their PLP research methodology, and others differ on how they reach this diagnosis. The IASP is clear, but there is a lack of data on the PLP diagnostic process [6, 8–10].

Thus, studies on PLP should always describe how it is investigated. This article reveals the PLP diagnostic process and characterisation used in the recruitment phase in the Brazilian research arm for a larger multicentre study called PLP, which evaluates the relationship between brain activity and PLP.

## 2. Objectives

The objective is to characterise PLP by describing the diagnostic process used in the recruitment phase of the PLP study and, secondarily, to determine predictors for the diagnosis of PLP.

## 3. Methodology

This cross-sectional study refers to the findings of the recruitment phase of the Brazilian arm of the PLP study, a randomised, multicentre, double-blind study conducted at the Institute of Physical Medicine and Rehabilitation (IMREA) and Lucy Montoro Rehabilitation Centre (São Paulo, Brazil) in collaboration with the Spaulding Rehabilitation Centre (Massachusetts, USA). It was approved by the Ethics and Research Committee CAAE:76850117.3.0000.0068 and registered as a clinical trial: NCT 02627495.

The inclusion criteria were patients aged over 18 years, with traumatic unilateral lower-limb amputation, with PLP for at least 3 months after full recovery from amputation surgery, having had at least 2 weeks of stable pain medication doses, and without any cognitive deficits.

The exclusion criteria were patients with any clinical decompensation; evidence of disease, trauma, or surgery in the central or peripheral nervous system; drug or alcohol abuse in the last 6 months; pregnancy; no prognosis for prosthetic gait; and having had exhibited suicidal ideation.

After signing the free and informed consent form, the subjects were evaluated for sociodemographic data (sex, age, and education), level of amputation, time since amputation, surgery, TSA, rehabilitation programme phase, and intensity of PLP using the visual analogue scale (VAS). The pain was classified according to the VAS in mild PLP, (0 > VAS > 40) mm, moderate PLP, (40 ≥ VAS > 70) mm, or severe PLP, (VAS ≥ 70) mm.

PLP was characterised according to the patient's free report, followed by an inventory of pain descriptors based on all sensory subgroups of the Full-Form McGill Questionnaire to help patients name what they felt. At the end, there was space for reporting additional sensations. All these descriptors were quantified for intensity by the VAS and by their weekly frequency.

The PLP research process followed the following script of questions:

(1). Do you suffer from unpleasant sensations in the missing leg?
(2) What is the intensity of this unpleasant feeling from 0 to 100? Demonstrate this intensity on the VAS scale.
(3) What unpleasant sensations do you feel in the missing part of the limb?
(4) What is the intensity of this unpleasant feeling from 0 to 100? Demonstrate this intensity on the VAS scale.
(5) How many times a week do you feel this unpleasant sensation?
  We will help you to describe everything you feel in the amputated part. We have a list of different sensations. We will recite them one-by-one. Please say if the word we recited defines what you feel.
(6) How much do you suffer (name the unpleasant feeling from the list) from 0 to 100? Demonstrate this intensity on the VAS scale.
(7) How many times a week do you feel (name of the unpleasant sensation)?
(8) Are there any other sensations that bother you? What are they like?
(9) What are their intensities from 0 to 100? Demonstrate their intensities on the VAS scale.
(10) How many times a week do you feel them?

The anamnesis methodology results in variables related to PLP descriptor numbers per patient, PLP descriptor-VAS and PLP-descriptor weekly frequency.

The sample distribution was nonparametric by the Shapiro–Wilk test (*p* < 0.05) and visual histogram analysis. The continuous variables, age (years), TSA (months), educational level (years of schooling), VAS (mm), and PLP descriptor-VAS and PLP-descriptor weekly frequency, were presented with medians and interquartile ranges. The categorical data, gender (women or men), ethnicity (Caucasian or Afro-South-American), amputation level (above or below the knee), rehabilitation programme phase (pre or prosthetic), PLP descriptor number per patient, and pain classification: mild, (0 > VAS > 40) mm, moderate, (40 ≥ VAS > 70) mm, or severe, (VAS ≥ 70) mm, were summarised as counts and proportions.

Spearman's correlation test was conducted for quantitative variables, followed by linear or logistic regression, when appropriate, to detect predictor relationships between the 24 descriptors of PLP and the variables of age, sex, education, ethnicity, level of amputation, and rehabilitation programme phase. All analyses were performed using the STATA® software version 15, Windows® (StataCorp, LP, College Station, TX), and a 2-tailed *p* < 0.05 was considered statistically significant.

## 4. Results

The study included 55 patients with 20–47 (10.63–52.93) months since traumatic unilateral amputation of the lower limb and who were predominantly male, Caucasian, young, with high-school education, above-knee amputees, in the preprosthetic phase of the rehabilitation programme, with a VAS for PLP of 60 (50–79.3) mm and with prevailing moderate-to-severe pain, as seen in Table 1.

Characterisation through the questionnaire showed that PLP is not a single painful symptom, but a set of symptoms for which patients have difficulty finding words to describe. PLP is a set of unpleasant sensations not identified as pain by patients, but as several descriptors that add up, overlap, and coexist.

The patients needed a median of 13 (6–20) different pain descriptors to show what they felt, and 30% needed more than 10. Besides not being a single painful symptom, five descriptors were continuous, seven times a week: burning, tingling, cooling, pulsations, and shocks. Other descriptors are added to this main pain, with a frequency of 3.94 (2.5–4.38) times a week.

The majority (70.91%) reports kinaesthetic painful sensations of the phantom limb, both voluntary and involuntary, frequently twisting (bending) in a static or dynamic, disruptive, seizure-like manner 5.5 (1–7) times per week. Itchiness, shock, tingling, and throbbing were other descriptors that followed in descending order of complaint, as shown in Table 2.

**Figure 1:**
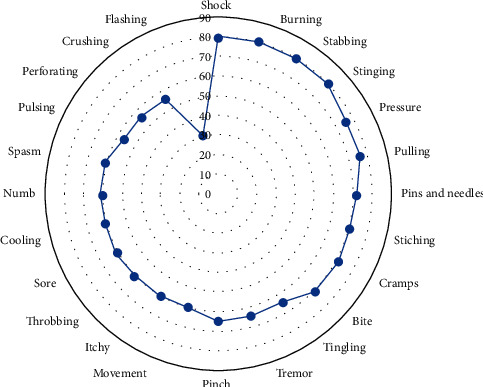
Intensity of descriptors measured by the VAS.

Figure 1 shows that, from all the descriptors, shock, burning, stabbing, and stinging were the most intense (median VAS = 80 mm). Except for flushing, all of them were evaluated as moderate-to-intense pain. Figure 1 shows the distribution of descriptors in descending order of intensity from the periphery to the centre.

**Figure 2:**
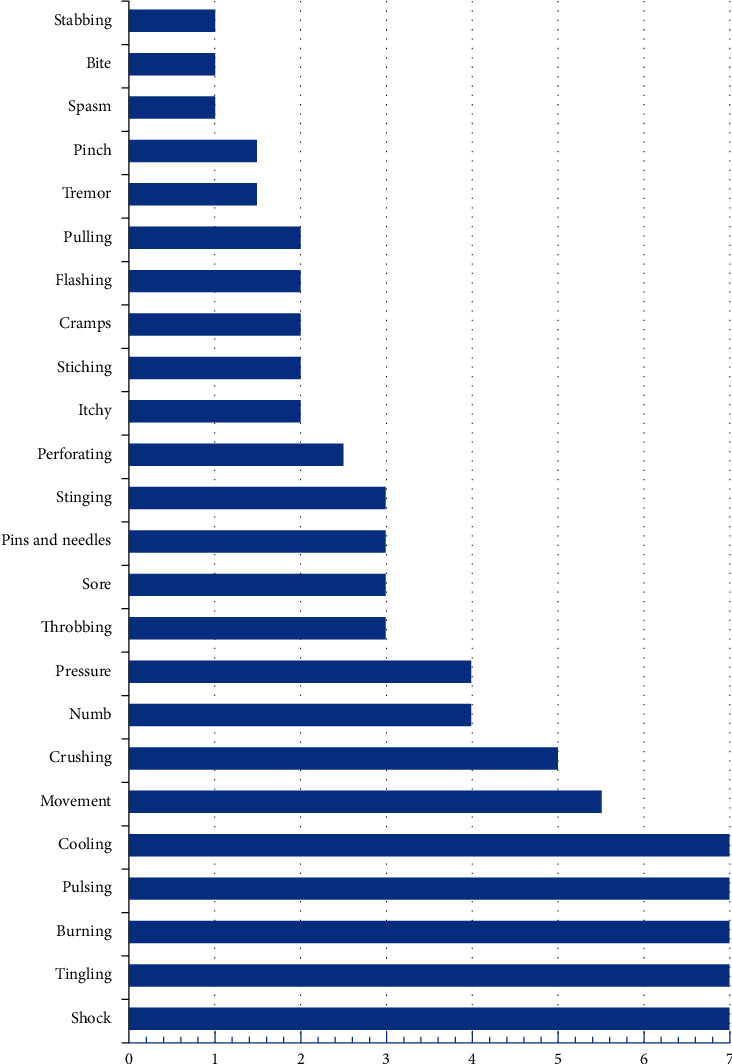
Distribution of the PLP descriptors' weekly frequency.

Each descriptor showed a different occurrence frequency and was either isolated or associated with other descriptors. Cooling, pulsing, burning, tingling, and shock occur daily, 7 days a week. The PLP-descriptor weekly frequency median is described in Table 2 and in Figure 2, which shows the weekly frequency of each descriptor in descending order.

To clarify the correlation between the PLP descriptor number per patient and the variables VAS, gender, educational level, amputation level, and age, Spearman's test showed a strong correlation between the education level and the number of descriptors reported by the patient (rho coefficients 0.2456, *r*^2^ = 0.64, *p*=0.034).

To find predictors of each PLP descriptor, logistic regressions were conducted having each of the 24 descriptors as dependent variables and sex, age, ethnicity, education level, amputation, and rehabilitation programme phase as independent variables. Spasm, tremor, throbbing, tingling, itchiness, numbness, and flushing could be predicted by the independent variables.  Table 3 shows the statistically significant results (*p* < 0.05) of these different analyses.

According to logistic regression, tingling is associated with the educational level (OR = 1.18 (1.01–1.39) *p*=0.04), rehabilitation programme phase (OR = 4.1 (1.27–13.21), *p*=0.02), and VAS (1.3 (1.02–1.65), *p*=0.03), in a statistically significant way (*p* < 0.05). Numbness has a 3.27 times higher likelihood of occurring in people with higher educational level and a 5.02 higher likelihood of occurring in the prosthetic phase. Flushing has a 4.2 times higher likelihood in above-knee amputations. Itchiness has a 1.20 times higher likelihood of occurrence in people with higher education. Spasm happens 3.1 times more often in above-knee amputees. Tremor and throbbing are 7.25 and 3.75 times more likely, respectively, to occur during the prosthetic phase, with statistical significance (*p* < 0.05). The descriptors could not be predicted by the variables sex, ethnicity, and age (*p* > 0.05).

## 5. Discussion

This study shows that PLP is not a single symptom, but is a set of symptoms that overlap and coexist and is always perceived in the region previously occupied by the patient's amputated limb. Each one of these sensations present with different intensities and frequencies, and patients commonly have trouble describing them in words. During the recruitment process, patients were noted to associate the word pain with nociceptive experiences that did not describe exactly what they felt as PLP. Thus, it was preferable to investigate the presence of PLP using the term “unpleasant sensation,” as defined by the IASP, to generate more reliable responses about what the patients felt [5, 6].

The sample centred on patients with traumatic unilateral lower-limb amputations without systemic diseases or diseases of the central or peripheral nervous system. Confounders that could interfere in afferences in the processing and interpretation of the central nervous system as well as in the efferent responses were excluded. This allowed for a better understanding of the symptomatology related exclusively to PLP since the current evidence of its origin is associated with neurogenic processes [11, 12].

Scientific literature relates PLP to maladaptive neuroplasticity, wherein the cerebral area responsible for the amputated area is invaded by neighbouring areas and new engrams, with the coexistence, in the same structures of engrams and processes of controls prior to the amputation superimposed with the conscious and unconscious learning of the new reality of the amputation. This involves a new pattern of visual, sensory, and motor afferences and afferents. This study confirms that the level of amputation is not associated with PLP in terms of quantity, intensity, or frequency; that is, the amount of afference lost by amputation may not be related to the whole neurogenic process that involves the appearance of PLP [13–17].

PLP is a new experience for patients who previously do not have any similar experience to aid in its identification [15]. Consequently, many patients do not recognise the unpleasant sensations they feel in the amputated part as pain, even though it is considered as PAIN by the IASP definition [5]. Thus, the investigation technique based on questioning using descriptors reported by the patients themselves may help to improve the diagnosis of PLP and, thus, the monitoring of treatments and the screening for new diagnostic processes that consider the central mechanisms of the generation of PLP [5, 6, 18–20].

Based on this experience, in the screening process for this research, when patients were asked “Do you feel phantom pain?,” most of them reported not feeling it because they associated the word pain with the descriptor score of the McGill Questionnaire which is present in only 45.45% of the sample in intensity and is not higher than the main descriptors reported by the patients. Thus, this question does not allow for the identification of PLP since the pain/sore sensation is not on the list of discomforts and priorities that the patients want to relieve. Therefore, this type of question contributes to the underdiagnosis and variability of PLP prevalence reported in the literature [2, 3, 7, 9, 10].

The literature does not indicate any instrument to diagnose or evaluate PLP. The McGill Pain Questionnaire, despite being widely used in studies about pain, is not validated for PLP [21]. This study shows that, of the 75 pain descriptors in the Full-Form McGill Pain Questionnaire, only 17 could reflect what the patients felt. The words most used to explain PLP were terms related to posture or positioning unpleasant of the amputated part (torsion, flexion, extension, and deformity) in a static or dynamic way such that the patients also did not identify them as the descriptor movement of the McGill. The results of this study show the importance of adjusting the guide questions for PLP research by including new descriptors, thus presenting an opportunity to create a diagnostic instrument based on what amputation patients feel and not on what professionals consider relevant [21].

Educational level, above-knee amputation, and prosthetic phase of the rehabilitation programme were associated with greater chances of the patient reporting more PLP descriptors. Higher education is associated with more employment opportunities, income, and access to reading, travel, and sightseeing, creating a repertoire of experiences and words that facilitate the understanding and expression of symptoms, which may explain why people with more years of education have PLP with more descriptors. More years of schooling enable a wider range of vocabulary, which further allows a more detailed expression of what one feels [22, 23]. The association of PLP with above-knee amputation can be explained by the larger area of the lost leg and consequently a larger orphan brain area, determining greater maladaptive neuroplasticity with a more chance of PLP [15, 18].

In the prosthetic phase of the rehabilitation programme, adaptation to the prosthesis begins, which requires new learning about preexisting engrams of the integral body (without the amputation), of the amputated body, and of the body with crutches in an environment of an orphan brain area under the influence of other brain areas and of the absence of the limb. This requires learning and incorporation of a new engram when walking with a prosthesis, which will need to coexist and cohabit brain areas in the process of reorganisation. Therefore, we believe this to be an important point that can generate additional suffering for the patient by recrudescence, intensification, or change of the PLP pattern, which then contributes to the rates of abandonment of prosthesis use [15, 24–26].

The existing diagnosis of phantom limb pain depends on the patient's report or subsequent medical records. The current study was also based on oral reports and subjective patient scores. The diagnostic methods used in this study were not basically different from previous studies, but reveal the need to use adequate descriptors to investigate the presence of PLP during anamnesis. The use of the term pain during the anamnesis does not reflect what the patient feels as PLP. The best terms refer to unpleasant sensations (as defined by the IASP) of movement, positioning, posture, cooling, pulsing, burning, tingling, shock, itchiness, or throbbing in the amputated part [5, 6, 21].

The authors recommend in this diagnostic process based on the patient's report that professionals replace “Do you feel pain in the amputated part?” to “Do you feel any movement, posture, positioning, cold, pulsation, burning, tingling, shock, itching, or throbbing in the amputated part?” to facilitate the meeting between the patient feel with the proper PLP diagnosis, as defined by the IASP.

The authors are concerned about study limitations related to cultural issues in the population and the absence of the control group. At this moment, there is no validation other than anamnesis for PLP diagnosis, as defined by the IASP [5, 6]. Traumatic amputation is a new experience in the patients' life, with no parallelism with painful pheromones that they have felt before.

The phantom pain experience, despite being defined as pain, is often not reported by patients as pain, but as an unpleasant sensory experience. In clinical practice, the answer to the question “Do you have phantom pain?” may not bring to the diagnostic discussion what they really feel, while the answer to the question “Do you feel any unpleasant sensations in the amputated part?” provides a diagnosis on what the IASP defines as pain.

This study is exploratory, and it is recommended to test the diagnostic power of these two anamnesis methodologies to know the potential of these techniques. Besides, comparing it with methods related to functional near-infrared spectroscopy (fNIRS) and functional magnetic resonance imaging (fMRI) for PLP diagnosis is the aim of the principal study which this study is part.

Pain duration was not considered for the study because time is not related to the diagnosis. Regardless of how long the patient feels, whether for a few moments or for many decades, if there is pain or unpleasant sensation in the amputated part, the diagnosis of PLP is confirmed, according to the IASP [5, 6]. The authors recommend additional studies about the correlation of pain duration with the quality of life in amputee patients.

## 6. Conclusions

PLP is not a single symptom. It is characterised by a set of different symptoms that add up, coexist, and overlap intensely in weekly cycles, without showing any parallelism to the patient's previous experiences. The best way to investigate the presence of PLP is to use expressions related to movement, posture, positioning, cold, pulsation, burning, tingling, shock, itching, or throbbing in the amputated limb.

## Figures and Tables

**Table 1:** Study population characterisation.

Age, median (IQR), years	39.5 (32–50)	
Women, *n* (%)	20	36.36%
Men	35	63.64%

TSA, median (IQR), months	20.47 (10.63–52.93)	
Caucasian	29	52.73%
Afro-South American	26	47.27%

Educational level, median (IQR), years	11 (8–21)	
Below the knee	26	47.27%
Above the knee	29	52.73%
Preprosthetic	31	56.36%
Prosthetic	24	43.64%

PLP, VAS (mm)	60 (50–79.30)	
Mild PLP, (0 > VAS > 40) mm	5	9.09%
Moderate PLP, (40 ≥ VAS > 70) mm	27	49.09%
Severe PLP, (VAS ≥ 70) mm	23	41.82%

*N* = number of observations; IQR = interquartile range; TSA = time since amputation; VAS = visual analogue scale; % = percentage.

**Table 2:** Phantom limb pain descriptor characterisation by the occurrence order, intensity, and weekly frequency of complaint.

PLP descriptor	*N*	%	VAS^*∗*^	IQR	*F*	IQR
Movement	39	70.91	60	(50–80)	5.5	(1–7)
Itchy	32	58.18	60	(40–80)	2	(0.25–7)
Shock	28	50.91	80	(57.5–90)	7	(0.5–7)
Tingling	28	50.91	65	(50–80)	7	(0.25–7)
Throbbing	26	47.27	60	(52.5–80)	3	(0.5–7)
Pain	25	45.45	60	(27.5–80)	3	(0.5–7)
Burning	21	38.18	80	(50–80)	7	(0.25–7)
Pins and needles	19	34.55	70	(55–80)	3	(0.5–7)
Pulsing	18	32.73	55	(40–80)	7	(0.5–7)
Stitching	17	30.91	70	(50–80)	2	(0.1–3)
Cramp	14	25.45	70	(50–80)	2	(0.25–6)
Tremor	14	25.45	65	(50–90)	1.5	(1–6)
Cooling	13	23.64	60	(50–70)	7	(1–7)
Numb	13	23.64	60	(50–80)	4	(1–7)
Pressure	10	18.18	75	(52.5–87.5)	4	(1–7)
Spasm	10	18.18	60	(27.5–60)	1	(0.5–1.75)
Perforating	8	14.55	55	(45–72.5)	2.5	(1–7)
Crushing	8	14.55	55	(50–82.5)	5	(0.5–7)
Bite	6	10.91	70	(47.5–70)	1	(1–3.5)
Stabbing	5	9.09	80	(70–80)	1	(0.1–1)
Pinch	4	7.27	65	(47.5–85)	1.5	(1–3)
Stinging	3	5.45	80	(65–85)	3	(2–5)
Flushing	3	5.45	30	(25–50)	2	(0.5–4.5)
Pulling	2	3.64	75	(72.5–77.50)	2	(1–2.5)

*N* = number of observations; % = percentage; VAS = visual analogue scale (mm); IQR = interquartile range; *F* = weekly frequency of descriptor occurrence.

**Table 3:** Logistic regression to detect variables associated with phantom limb pain descriptors.

Descriptor (outcome)	Independent variable	OR	CI 95%		*p*
Tingling	Educational level	1.18	1.01	1.39	0.04
Tingling	RPP	4.1	1.27	13.21	0.02
Tingling	PLP intensity by the VAS	1.3	1.02	1.65	0.03
Numb	Educational level	3.27	1.2	8.96	0.02
Numb	RPP	5.02	1.3	19.31	0.02
Flushing	Amputation level	4.2	1.36	12.96	0.01
Itchy	Educational level	1.20	1.02	1.41	0.03
Spasm	Amputation level	3.1	1.14	8.46	0.03
Tremor	RPP	7.25	1.9	27.64	0.00
Throbbing	RPP	3.75	1.19	11.77	0.02

OR = odds ratio; CI95% = 95% confidence interval; *p* = *p* value; RPP, rehabilitation programme phase.

## Data Availability

The raw data used to support the findings of this study are available from the corresponding author upon request.

## Additional Points

Diagnosis is the key point in enhancing PLP prevalence and incidence rates. Understanding PLP as pain sets enhances diagnosis rates. Understanding what patients feel as PLP is the first step in achieving good communication techniques to improve diagnosis rates.
